# A Review of Respiratory Anatomical Development, Air Flow Characterization and Particle Deposition

**DOI:** 10.3390/ijerph17020380

**Published:** 2020-01-07

**Authors:** Mohammad S. Islam, Gunther Paul, Hui X. Ong, Paul M. Young, Y. T. Gu, Suvash C. Saha

**Affiliations:** 1 School of Mechanical and Mechatronic Engineering, Faculty of Engineering and Information Technology, University of Technology Sydney, Ultimo, NSW 2007, Australia; Mohammadsaidul.islam@uts.edu.au; 2 Australian Institute of Tropical Health and Medicine, James Cook University, Mackay, QLD 4741, Australia; gunther.paul@jcu.edu.au; 3 Respiratory Technology, Woolcock Institute of Medical Research and Discipline of Pharmacology, Faculty of Medicine and Health, The University of Sydney, 431 Glebe Point Road, Glebe, NSW 2037, Australia; Ong.hui@sydney.edu.au (H.X.O.); paul.young@sydney.edu.au (P.M.Y.); 4 School of Chemistry, Physics & Mechanical Engineering, Queensland University of Technology 2 George Street, GPO Box 2434, Brisbane, QLD 4001, Australia; yuantong.gu@qut.edu.au

**Keywords:** airflow, particle transport, particle deposition, particle-particle interaction, Euler-Lagrange method, Euler-Euler approach

## Abstract

The understanding of complex inhalation and transport processes of pollutant particles through the human respiratory system is important for investigations into dosimetry and respiratory health effects in various settings, such as environmental or occupational health. The studies over the last few decades for micro- and nanoparticle transport and deposition have advanced the understanding of drug-aerosol impacts in the mouth-throat and the upper airways. However, most of the Lagrangian and Eulerian studies have utilized the non-realistic symmetric anatomical model for airflow and particle deposition predictions. Recent improvements to visualization techniques using high-resolution computed tomography (CT) data and the resultant development of three dimensional (3-D) anatomical models support the realistic representation of lung geometry. Yet, the selection of different modelling approaches to analyze the transitional flow behavior and the use of different inlet and outlet conditions provide a dissimilar prediction of particle deposition in the human lung. Moreover, incorporation of relevant physical and appropriate boundary conditions are important factors to consider for the more accurate prediction of transitional flow and particle transport in human lung. This review critically appraises currently available literature on airflow and particle transport mechanism in the lungs, as well as numerical simulations with the aim to explore processes involved. Numerical studies found that both the Euler–Lagrange (E-L) and Euler–Euler methods do not influence nanoparticle (particle diameter ≤50 nm) deposition patterns at a flow rate ≤25 L/min. Furthermore, numerical studies demonstrated that turbulence dispersion does not significantly affect nanoparticle deposition patterns. This critical review aims to develop the field and increase the state-of-the-art in human lung modelling.

## 1. Introduction

Better knowledge of air exchange processes in breathing, coughing, and sneezing are important for improved respiratory health risk assessment. A wide range of published literature has increased the knowledge of airflow characterization and particle transport in human lung. Understanding particulate matter transport, movement, and deposition in the terminal airways is the primary step to predicting and helps preventing respiratory diseases. Various human activities like combustion of fossil fuels in vehicles, industrial processes, thermal coal combustion, biomass burning, and many other sources produce a significant amount of particulate matter (PM) [1]. The uptake of PM through nasal or oral inhalation follows a complicated transport through the irregular dichotomous branching airways. Most of the inhaled pollutant may settle or escape the respiratory tract during the breathing process by different mechanisms. Some particles may be absorbed through the epithelium and initiate a cascade of inflammatory processes that lead to various respiratory diseases [2]. The toxicity and carcinogenic potential of PM on respiratory health are based on their residence time in the different regions of the airway. The soluble and insoluble aerosols can be much more harmful in the terminal airways of the different lobes depending on their toxicity. The abnormal cilia and absence of mucociliary clearance at the terminal and alveolar airways cannot clear the toxic particle [3]. Studies over the past few decades improved the knowledge of the airflow and particle transport in the idealized airway. In recent years, technological advancement helped to construct the realistic CT-based anatomical model. A wide range of studies has employed different calculation methods and anatomical models for airflow and particle transport in human airways. This study aims to critically analyse the recent development and summarize the available literature of the field. The objectives of the present review are (i) to examine the current state of anatomical lung model development (realistic and non-realistic); (ii) to evaluate airflow characterisation in a whole lung model; (iii) to compare microparticle transport and deposition (TD) in upper and lower airways predicted by different models, including the effects of ultrafine particle TD in extra-thoracic, and tracheobronchial airways.

## 2. Anatomical Lung Model Development

### 2.1. Conventional Non-Realistic Lung Model

Quantitative information contained in a human lung anatomical model such as the number of generations and branches, dimensions, shapes, surfaces, geometric correlation, and arrangement is an important factor to consider in respiratory health risk assessment. Hales [4] first investigated the airflow in a calf lung and calculated the total inner surface of the lung. Aslett, Hart and McMichael [5] described lung volume and subdivisions, in a study of 66 adult male subjects. Briscoe and Dubois [6] calculated resistance and airway dimensions for different age lungs. The study established a relationship between inhalation resistance and lung volume. Human lung branching patterns are highly complicated and differ based on age, sex, and pulmonary diseases. The most simple lung model was proposed by Weibel [7], which was symmetrical with a branching pattern of equal diameter ratio. However, the model is far from a realistic branching pattern. A more realistic branching pattern was subsequently proposed by Horsfield, Dart, Olson, Filley and Cumming [8] for a young male; it illustrated that daughter branches were not identical. The authors also assumed that airway generation terminates before reaching its 23rd generation, and numbered the generations in the opposite order. Raabe, Yeh, Schum and Phalen [9] then developed a more realistic and asymmetric anatomical model for a replica of human casts by considering the branching and gravitation angles. In a five lobe model proposed by Yeh, Schum and Duggan [10], the individual lobes’ branching patterns were measured from the silicon rubber cast of a rat model. An asymmetric “typical path model” was then proposed by Yeh and Schum [11] based on the previous study. The typical path model considered the angle of inclination due to gravity along with other morphological parameters. Finally, a fully stochastic multiple path (SMP) model was developed by Koblinger and Hofmann [12]. In the SMP model, inhaled particles followed a random path out of millions of possible pathways. Variability in diameter and length of the airways in the SMP model were defined by lognormal frequency distribution. While this development has increased the knowledge of human lung branching patterns, cross-sectional area of the branching tube, branching angle and other parameters of the lung morphology, the smooth surfaces of these previous models of the tracheobronchial airway and bifurcating curvature were far from the realistic computed tomography (CT) scan based anatomical models.

### 2.2. Realistic Lung Model

**Figure 1:**
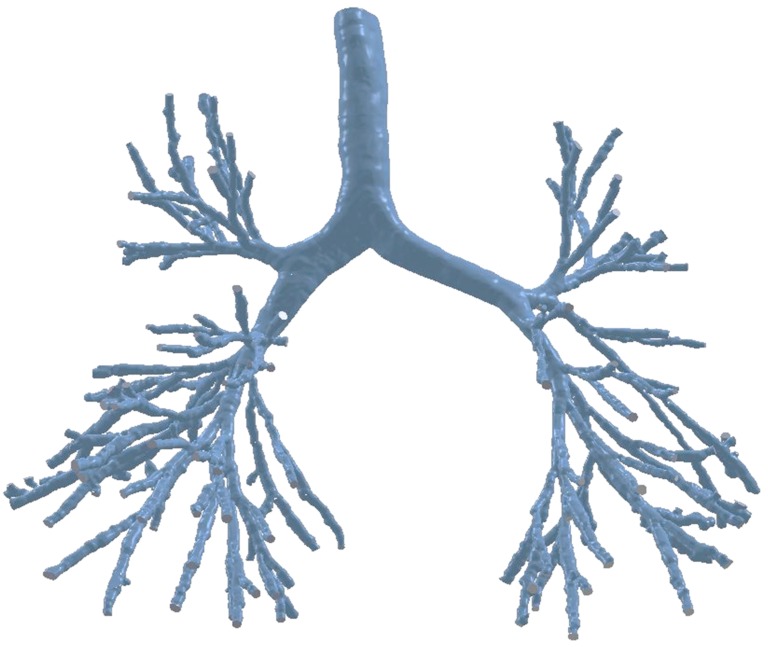
Human lung anatomical model from high-resolution CT-data.

Advancements in segmentation algorithms and high-resolution imaging techniques have helped overcome the lack of a realistic anatomical model for human lung modelling. Different segmentation techniques and software such as AMIRA [13,14], and DOCTOR [15] have been used to construct a 3-D anatomical model. According to literature, Sauret, Goatman, Fleming and Bailey [16] first developed an algorithm to calculate the dimensions and topology of human lung pathways from CT-scan data. However, the low resolution of the CT images restricts the modelling of the conducting airways up to 9 generations [17]. Later, Garrity, Segars, Knisley and Tsui [18] developed a 3-D airway anatomical model of up to five generations and extended the remaining airways based on a mathematical algorithm. Different imaging techniques, CT-Scan DICOM [19,20], or MRI [18] have been used to generate 3-D airway models. Table 1 summarises currently available realistic anatomical models of respiratory airways. However, all of these published models consist of a limited number of airway bifurcations. Recently, Islam, S. C. Saha and Young [21] constructed a 3-D realistic lung anatomical model for the healthy human cast with a large number of bifurcating airways (Figure 1). The DiCom images of 55-year-old man used for the 3-D anatomical model. The highly asymmetric lung anatomical model consists of the first 16-generations of airway branching, with the possibility to expand the entire branching pattern; as well as a highly asymmetric airway tracheobronchial wall surface. Airflow characterization to the bifurcating airways of the lung is important from both a numerical and experimental point of view, and the next section will discuss airflow characterization in the human lung airways.

## 3. Air Flow Characterization

### 3.1. Extrathoracic Region

Extra-thoracic airways (nasal passages, mouth-throat, larynx, pharynx) are the gateway of the respiratory system as inhaled and exhaled air passes through the nose or the mouth. The nasal passages or mouth throat structure is anatomically complex. Mouth and nasal cavity operate and are located in parallel, and the nasal cavity has two parallel passageways for air transport [33]. The physiological functions of the nasal cavity include respiration, humidification, heat exchange, filtration, nasal resistance, nasal cycle, and others [34]. Respiration is the most important physiological function of the nose for all creatures. The tortuous anatomical shape of two parallel nasal passages produces airflow resistance during air transport into the respiratory system. About 67% of total airway resistance is produced by the nasal passages [35]. Local obstruction at the nasal cavity significantly influences the intrapharyngeal pressure [36], which eventually leads to catastrophic failure of the lung [35]. Mouth breathing is the other possible way for human respiration. During heavy activities or under different diseases conditions, mouth breathing is more effective to transport sufficient air to the respiratory system.

The extrathoracic or nasopharyngeal region of the human lung includes the nose (anterior nasal passage and posterior nasal passage), mouth, larynx, and pharynx. A precise understanding of the airflow characterization in the extrathoracic airways is the primary step when modelling pathogenesis of respiratory diseases. A wide variety of in slilico [23,33,37,38,39]; refs. [40,41] and experimental models [42,43,44] have been considered to analyse the airflow pattern in the extrathoracic region. A commonly used model for airflow characterization in the extrathoracic airways are either non-realistic idealized model [23,45,46,47] or realistic CT-based models [15,23,39,48,49,50,51]. Some of the published literature considered laminar flow in this region [40,52,53] whereas, most of the studies are based on turbulent flow [42,45,46,54]. In reality, airflow becomes locally turbulent at the glottis region of the extrathoracic airway depending on the inhalation rate. Zhang and Kleinstreuer [55] reported that turbulent fluctuation happens past the throat and can continue downstream to the first three generations of the upper airways. Chen, Feng, Zhong and Kleinstreuer [56] reported that air flow becomes turbulent near the mouth cavity at 15–45 L/min flow rate. Different turbulent models, Reynolds-averaged Navier–Stokes (RANS) [57]; [58,59], k-ϵ [60,61], k-ω [39,62], Large Eddy Simulation (LES) [63,64,65,66] have been used for airflow characterization in the oral airways of the lung. RANS turbulence model solves the time-averaged equations of motion which is computationally less expensive. On the contrary, LES simulation employs different sub-grid models for smaller eddies, which is more accurate and computationally expensive. LES model can analyse the transitional behaviour of the fluid flow. All RANS based turbulence models (standard k-ϵ, RNG k-ϵ, realizable k-ϵ, standard k-ω) calculate the turbulence viscosity differently. The turbulence viscosity term of standard k-ϵ, RNG k-ϵ, and realizable k-ϵ turbulence model is µ_T_ = f(ρk^2^/ϵ). The viscosity term for standard k-ω and SST k-ω turbulence model is µ_T_ = f(ρk/ω). Further to that, Zhang and Kleinstreuer [67] investigated the flow regime in locally constricted conduits and compared different turbulent models. Their study reported that a proper low Reynolds number (LRN) turbulence model could accurately predict not only the transitional and turbulent flow regime, but also the laminar flow pattern. The comparative study concluded that the LRN turbulent model is better than any other turbulent model to predict transitional airway flow behavior.

**Figure 2:**
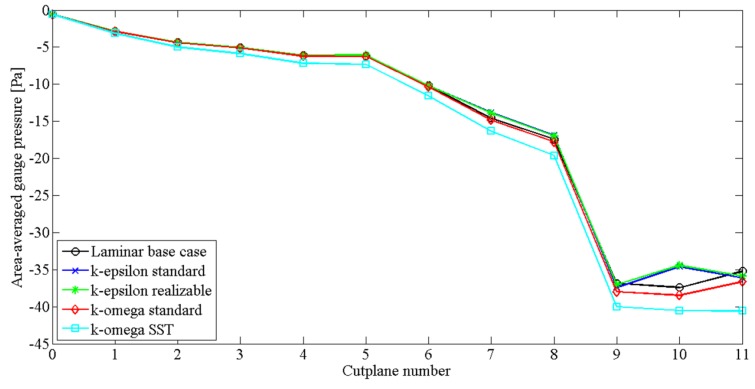
Comparison of area-averaged gauge pressure for the laminar base-case and four different turbulent models [61].

Mihaescu, Murugappan, Kalra, Khosla and Gutmark [68] investigated obstructive sleep apnoea in a realistic pharyngeal airway by employing different turbulent models. This study used a digital pharyngeal model which was developed using a maximum narrowing at the position of the pharynx. The study reported that the LES model was a better option for transitional flow modelling than RANS; and k-ω was slightly better than the k-ϵ turbulent model. The RANS turbulent model cannot quantify transitional behavior of flow separation effects. Furthermore, Heenan, Matida, Pollard and Finlay [69] concluded that RANS cannot predict viscous effects for lower Reynolds numbers (Re). Riazuddin, Zubair, Abdullah, Ismail, Shuaib, Hamid and Ahmad [70] used the k-ω SST turbulent model to analyze flow patterns at the nasal cavity, and the results showed a more accurate correlation with experimental data. Recently, Chen, Feng, Zhong and Kleinstreuer [56] also reported that the transition SST turbulence model was better than the RANS model for transitional flow analysis. Moreover, a numerical investigation of Aasgrav, Johnsen, Simonsen and Müller [61] reported the comparison of area-averaged gauge pressure for laminar and different turbulent cases (Figure 2).

### 3.2. Tracheobronchial Region

Airflow characterization during inhalation and exhalation through the non-dichotomously bifurcating airways is the primary step to predict particle transport in human lung. Flow features in the tracheobronchial airways are highly complex due to the non-uniform and complex anatomical structure of the pathways [15]. Different physiological factors (anatomical model, transient behavior of the flow) influence the flow pattern in the tracheobronchial region [19]. In an earlier study, Carrier [71] measured airflow in human lung during running and walking conditions. The study illustrated that lung ventilation drops significantly during movement. A wide range of studies have been conducted for airflow characterization in the tracheobronchial region by considering regular and symmetric [72,73,74,75] and asymmetric smooth airway [63] models. Airflow in the small pathways of the tracheobronchial regions shows a highly complex phenomenon. Soni, Lindley and Thompson [76] investigated how non-planarity and asymmetry affects secondary flows in the narrow tubes. The 90^°^ out-of-plane non-planar and asymmetric model shows a highly complex vortex pattern, whereas the symmetric planar model shows a symmetric vortex pattern. Schroter and Sudlow [77] analyzed the flow profile for a single bifurcation model and reported that the flow was steady for a Reynolds number of 1500. However, Gatlin, Cuicchi, Hammersley, Olson, Reddy and Burnside [78] reported that air flow in an asymmetric tubular bifurcation is laminar for Reynolds numbers less than 1000. Subsequently, Soni, Thompson and Machiraju [79] reported that secondary flow patterns become more complex further down the pathways due to branching and non-planarity. Furthermore, Bernate, Geisler, Padhy, Shaqfeh and Iaccarino [66] found the flow was chaotic at the most distal pathways and the calculated Reynolds number of those airways was as low as 300. However, a hot-wire anemometer experimental study reported fluid flow variation in the tracheobronchial airways with a Reynolds number as low as 100 [80]. The experimental study concluded that turbulent eddies created the unsteadiness at the central airway and convected to the downward airways. Zhang and Kleinstreuer [55] reported that the local anatomical structure (bifurcation area) influenced unsteady flow formation. The following study also concluded that turbulent instability which occured at the extrathoracic region continued into the trachea and first three generations. Olson [81] illustrated that unnatural sharp curvature of the airway influenced secondary flow. A number of studies [82,83] reported that the Reynolds number fell with an increase of branching angle, which shows that the anatomical model influences the flow pattern. The anatomical structure of the human lung impacts the flow field [23,84] and realistic lung airways exhibit highly complex uneven curvatures [26]. Recently, a realistic CT-based anatomical model [15,66,85,86,87,88,89,90,91] was used to characterize the transitional behaviour of airflow in human bifurcating pathways. In the realistic model, turbulent laryngeal jet follows turbulent flow [23] and the inspiratory flow field is found to be much more complex than in the symmetric lung model [20]. For a realistic turbulent model, the axial velocity at the main airway is more uniform in the airway centre than in the laminar case [20], and the axial flow field is significantly weaker at the right upper lobe [20]. Despite the improvements in realistic anatomical models, the appropriate boundary conditions of flow characterization are still not agreed. Different approaches, such as constant pressure [63,92,93], impedance modelling [94], or mass flow rate [95,96] are used for airflow modelling. However, cyclic breathing conditions [19,97] provide a good understanding of the air flow pattern in human lung. Walters and Luke [98] used constant zero pressure at the regional outlets and a stochastic coupling approach to set the pressure at truncated outlets. The fractional values of truncated and regional pathways from the steady-state solution were used as the initial conditions for unsteady breathing. In reality, there is a small pressure difference at the outlet of the terminal airways. However, none of the published literature considered a whole lung model by considering entire pathways. Thus, an open outlet (zero pressure) boundary condition was sufficiently accurate to predict the flow field.

**Figure 3:**
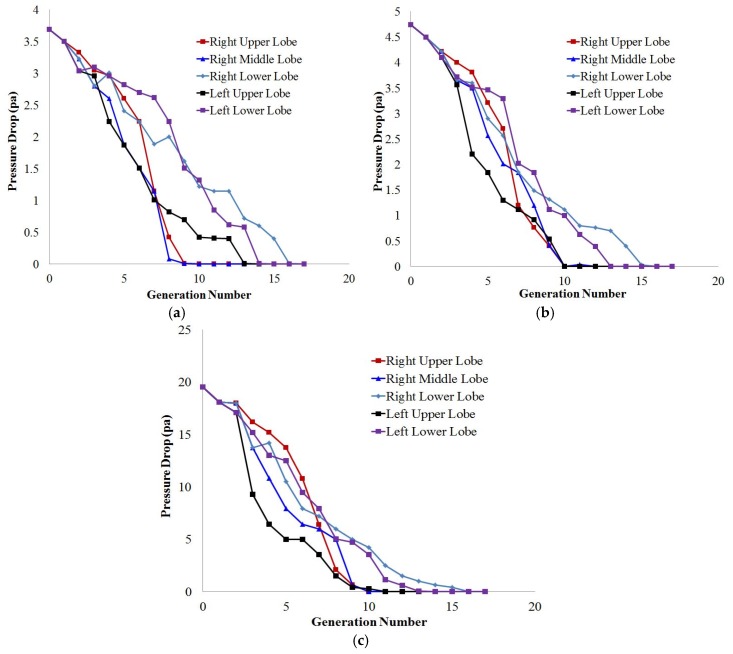
Pressure drop along a selected path line of lobes. (**a**) 7.5 lpm, (**b**) 9 lpm, and (**c**) 25 lpm [99].

Gemci, Ponyavin, Chen, Chen and Collins [63] investigated the nature of the secondary vortices flow in the terminal bronchioles of a large-scale 17-generation model. The large-scale model considered highly asymmetric airways with possible entire branches. The airflow study reported that secondary flow pattern varied at the different bronchioles due to the variation of the anatomical branching patterns. Recently, Islam, Saha, Sauret, Gemci, Yang and Gu [99] calculated flow distribution along five different lobes of a 17-generation model and found that the flow distribution percentage at the right lung was 1.5 times higher than the left lung (Table 2). More specifically, the flow distribution percentage at the lower lobes of the right and left lung was found higher than the remaining lobes. The numerical approach of Islam, Saha, Sauret, Gemci, Yang and Gu [99] also calculated pressure drop along the differently selected path line of five different lobes and pressure drop for the 17-generations shows a non-linear trend along the different lobes (Figure 3). Figure 3 reports that the pressure mostly fluctuate at the upper airways of the various lobes. The highest pressure drop is observed at the left upper lobe for all flow rates and a drastic pressure change is occurred at the third and fourth generation of the left upper lobe.

The human lung interacts with its environment by breathing, and aerosols are inhaled into the lung through gas exchange. Particle–lung interaction is important for respiratory health risk assessments, such as in occupational or environmental health settings; and in therapeutic study. The next section of this review will therefore discuss particle TD in the respiratory airways.

## 4. Particle Transport and Deposition

### 4.1. Micro-Particle Transport and Deposition

#### 4.1.1. Extrathoracic Region

A wide range of in vivo, in vitro and computational fluid dynamics (CFD) studies have been performed for flow-field analysis and microparticle TD in the extrathoracic airways [15,100,101,102,103,104,105]. The critical understanding of the local and regional micro-size aerosol deposition is important for assessing pulmonary health risk and drug deposition efficacy. The local deposition prediction in an extrathoracic model is important for tracheobronchial deposition prediction as the respiratory airway extends from the mouth-throat to the trachea. The highly complex anatomical structure of the upper airways plays a significant role in the local deposition of aerosols in the extrathoracic airways. A variety of studies [45,58,106] has been conducted to predict the aerosol particle deposition in the extrathoracic region of the human lung using idealized anatomical models of circular or elliptic or constant diameter [107,108]. Recently, realistic CT/MRI based [109,110,111] anatomical models have been used to predict the local deposition in the extrathoracic airways. Xi and Longest [62] conducted a comparative study for local deposition in the mouth throat to larynx for different anatomical (realistic, circular, elliptic, fixed diameter) models and concluded that the CT-based realistic model provided the most accurate prediction of the experimental measurements. The tortuous shape of the nasal cavity and highly complex extrathoracic airways induces turbulent flow even for low Reynolds numbers, significantly influencing the local deposition hot spot. A group of experimental measurements [112,113] and CFD approaches [114,115] has been performed to identify the particle deposition hot spot in the extrathoracic airways. Farhadi Ghalati, Keshavarzian, Abouali, Faramarzi, Tu and Shakibafard [114] investigated the airflow effects on aerosol particle transport in the extrathoracic airways of 24-year old women and the study reported four major locations for microparticle deposition. The next section will discuss the particle TD in the tracheobronchial region of human lung.

#### 4.1.2. Tracheobronchial Region

A wide range of studies has been conducted for flow field characterization and particle TD in the tracheobronchial airways [101,116,117,118,119,120,121,122,123,124,125,126]. Almost all of these studies consider non-realistic symmetric [127,128,129,130] and non-realistic asymmetric [88,93,131] anatomical models for airflow analysis and particle TD. Table 3 summarises a list of available published particle deposition models. A comprehensive comparison among different available models (semi-empirical, trumpet, single path, multiple paths stochastic) and the theoretical prediction of particle deposition has been conducted by ICRP and Protection [132]. This study increased the theoretical understanding of the anatomy and physiology of human lung, and radioactive particle transport in the respiratory tract. Triple bifurcation lung models are mostly used for air flow and particle deposition studies. Kleinstreuer, Zhang and Li [75] performed an airflow and particle deposition study for a triple bifurcation model and reported high inertial impaction of microparticles to form the deposition hot spot at the carinal angle.

It is evident that airways usually expand more during breathing under light and heavy physical activities conditions [139,140] compared to normal breathing. A study on pig airway also showed a very small change in the tracheal measurement during a normal breathing condition [141]. Later, Noble, Jones, Needi, Cairncross, Mitchell, James and McFawn [142] reported that normal inspiration does not influence the airway resonance in their in vitro study. However mechanical stretch of the airways occurs during deep inhalation. Despite this important physiological change of the airways, almost all of the previous studies did not consider the airway motion to account for airflow and particle transport. Recently, a CFD study with a novel moving mesh for the airway wall was conducted by Mead-Hunter, King, Larcombe and Mullins [134] for a triple bifurcation model. The cumulative deposition fraction for the upper airway model demonstrated similar deposition fraction for steady flow and a moving mesh case. However, the oscillating flow case showed significant discrepancy with the moving mesh case.

A large-scale, nine-generation airway model was considered to identify the thermal effects of air on particle deposition and found a negligible impact on particle deposition [143]. Longest, Tian, Khajeh-Hosseini-Dalasm and Hindle [102] on the other hand, developed an individual stochastic path (SIP) model and studied the deposition pattern for bifurcation 1 to 15 (B1–B15). The model only considered a single bifurcation for each generation and predicted the deposition pattern. A comprehensive comparison of analytical and CFD models has been conducted for a triple bifurcation model up to the first 16 generations [73]. The authors have considered a different triple bifurcation model (G1–G3, G3–G6, and so on) up to 16 generations and reported that airway bifurcation rotation had a minor effect on particle deposition. Later, Zhang, Kleinstreuer and Kim [138] investigated the global and local deposition pattern in a triple bifurcation tracheobronchial unit and reported that local bifurcation and flow pattern significantly influence the deposition pattern. All of the above-mentioned in silico models considered a non-realistic smooth tracheobronchial wall surface, which is far from the realistic anatomical model. Table 4 shows the list of available literature on airflow and particle TD in the human respiratory tract.

In recent years, a number of realistic anatomical models have been developed for the extrathoracic [22,23] and tracheobronchial [25,26,29] region of the lung. Ref. [19] developed an anatomical model of up to 15 generations from CT-scan data. However, only a few of them considered particle TD in their study.

Laminar and different turbulent models are used to predict the particle TD in the tracheobronchial airways. A number of studies considered laminar flow [51,77,144] for the prediction of local deposition in the tracheobronchial airways. However, the flow becomes locally turbulent in the glottis region of the lung that significantly affects the microparticle deposition pattern. Zhang and Kleinstreuer [55] reported that turbulent dispersion which arises at the extrathoracic region might persist up to the first few generations for a flow rate greater than 30 lpm.

In reality, during inhalation, particles can collide with each other, although most of the published literature did not consider particle–particle collision in their study. However, if the particle suspension entering the tracheobronchial airways is diluted, then direct particle–particle interactions can be neglected. The interaction between particles plays an important role in particle TD in human lung [145]. Cundall and Strack [146] have proposed a soft sphere approach to consider particle–particle interaction. Chen, Zhong, Zhou, Jin and Sun [147], investigated the CFD-Discrete Element Method (DEM) calculation for particle transport in the lung airway, and they concluded that the particle inlet position might affect the particle trajectory. Recently, Feng and Kleinstreuer [148] have investigated particle–particle interaction for a triple bifurcation airway model. The dense discrete phase-DEM model investigated diluted and dense particle TD in different sets of triple bifurcation models. The two-way DDPM-DEM model showed slightly lower deposition efficiency (DE) compared to the one-way model. However, no published literature is currently available investigating particle–particle interaction in a CT-based realistic airway model. Hence, this study performed a DPM-DEM case study for particle–particle interaction in the realistic upper airways.

The Spring-Dashpot collision law is used to calculate the particle contact forces. In the case of the linear spring collision law, two collidin particles are considered. A unit vector (${\vec{e}}_{12}$) is defined for the two particles from the centre of the particle 1, and the centre of the particle 2 as

(1) $${\vec{e}}_{12}=\frac{\left(x_{2}-x_{1}\right)}{\left\|x_{2}-x_{1}\right\|}$$

where *x*_1_ and *x*_2_ are the position of the particle 1, and the particle 2 respectively. The overlap (*δ*) between two particles during the contact which is must be less than zero is defined as

(2) $$\delta =\left\|x_{2}-x_{1}\right\|-(r_{1}+r_{2})$$

where $r_{1}$ and $r_{2}$ are the radius of the particle 1 and particle 2 respectively.

For Spring-Dashpot collision law, a dashpot term (*η*; 0 < *η* ≤ 1) is defined with spring constant (K) and the coefficient of restitution. For the force calculation during the particle collision, the expressions for the loss factor and so-called reduced mass of the two particles are defined as

(3) $$f_{loss}=\sqrt{\pi^{2}+\ln^{2}\eta }\;m_{12}=\frac{m_{1}m_{2}}{m_{1}+m_{2}}$$

The collision timescale (*t_coll_*) of the particle and the damping coefficient (*γ*) are defined as

(4) $$t_{coll}=\sqrt{\pi^{2}+\ln^{2}\eta }\sqrt{\frac{m_{12}}{K}}$$

(5) $$\gamma =-2\frac{m_{12}\ln\eta }{\sqrt{\pi^{2}+\ln^{2}\eta }\sqrt{\frac{m_{12}}{K}}}$$

The relative velocity between particle 1 and particle 2 can be defined as

(6) $${\vec{v}}_{12}={\vec{v}}_{2}-{\vec{v}}_{1}$$

here ${\vec{v}}_{1}$ and ${\vec{v}}_{2}$ are the velocity of the particle 1, and particle 2 respectively.

The present model used the so called ‘soft sphere’ approach to accounts the force that results from the particle–particle collision. For the soft sphere approach, the forces from the inter-particle collision are determined by the deformation or overlap between the two particles. The particle motion equation is integrated over time to capture the interaction of the particle. The normal spring constant value for the soft sphere approach can be estimated by the collision time, restitution coefficient, and the relative velocity of the colliding particle. The spring constant value influences the time step of the simulation. For the large spring constant value, the simulation needs smaller time steps to calculate the particle–particle interaction accurately.

**Figure 4:**
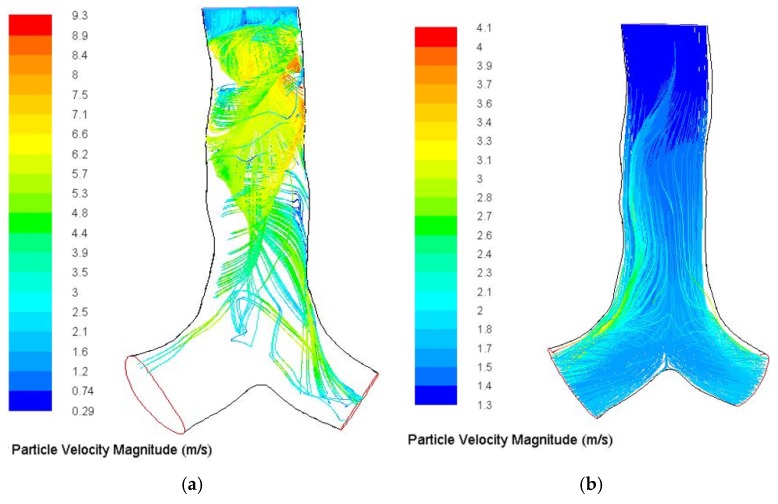
10-µm diameter particles track streamlines in the upper airways with and without collision, (**a**) with interaction and (**b**) without interaction. Particle traces are based on particle velocity magnitude.

The tracking streamline of 10-µm diameter particles with and without particle–particle interaction is shown in Figure 4. Figure 4a shows particle traces colored by particle velocity magnitude. The overall particle trace demonstrated complex particle trajectories at the top of the trachea, and eventually depicts strong particle–particle interaction for 10-µm diameter particles. Figure 4b on the other hand shows particle traces of 10-µm particles without particle–particle interaction. Particle–particle interaction therefore eventually increases the overall deposition in the upper lung region.

To show particle deposition in the bifurcating airways, different deposition criteria or assumptions were used. Li, Kleinstreuer and Zhang [149] and Nazridoust, Asgharian and Asgharian [150] assumed that if the center of the inhaled particle came within less than the radius of the particle from the airway surface, then the particle would be considered as deposited. However, in reality, the airway wall consists of a mucus and periciliary fluid layer [151], which is viscous [152]. Inhaled particles are trapped at the airway wall as soon as the particle touches the mucus surface. A wide range of studies [13,26,116,153,154] has considered a trapped boundary condition to calculate the deposition pattern in human lung.

#### 4.1.3. Alveolar Region

Some respiratory diseases mainly occur at the terminal airways as fine particles can travel to the very end of the deeper airways. Based on residence time, pollutant particles can cause respiratory epithelium damage, inflammation and may also lead to tumor formation. Parkash [155] and Pityn, Chamberlain, King and Morgan [156] reported that respiratory damage mainly occurs at a specific position of the different lobes. More specifically, Parkash [155] found that carcinomas develop in the right lung more often than the left lung. Only a few theoretical and CFD studies have been conducted for lobar deposition prediction. Lobar deposition fraction data for five different lobes reported in a mathematical scheme found that the deposition pattern significantly depended on lung morphometry [157]. Only the analytical multiple-path particle dosimetry model (MPPD) [157] could predict the lobar deposition for an asymmetric lung model. Recently, the CFD approach of a stochastic individual path (SIP) model [95] predicted the lobar deposition for five different lobes of the human lung. However, the acinar region is the most important part of human lung and detailed information for acinar geometry is not available. A number of studies [7,158] reported that the acinar structure of a human cast varies from person to person and the alveolar shape is relatively spheroid [158]. Recently, digital images have been used to construct an alveolar model and found that alveolar topology structure is highly irregular [159]. There are only a few studies [160,161,162] that performed numerical investigation of particle deposition in the alveolar region of human lung. The simplified numerical model of Talaat and Xi [160] demonstrates that alveolar structure and particle diameter effects on airflow and deposition. The study concluded that alveolar movement influences particle deposition in the alveolar region. The next section of this review will discuss ultrafine particle TD in the extra-thoracic and tracheobronchial airways.

### 4.2. Ultrafine Particle Transport and Deposition

#### 4.2.1. Extra Thoracic and Tracheobronchial Region

Different sources, such as industrial combustion, release a significant amount of nanoparticles into the atmosphere. After release, particles may further develop through condensation of the gas clusters, or coagulation with other particulates. Nanomaterial products from industrialization and the increased popularity of these nanomaterial products also emit a significant amount of ultrafine particles. The incomplete combustion process is used to form polycyclic aromatic hydrocarbons (PAH), which are a compound of more than 100 different chemicals [1,163]. The pollutant PAH can exist in different forms (volatile, semi-volatile) and they may combine with another pollutant particle in the atmosphere.

For this reason, nanoparticles can be more hazardous compared to microparticles and may contribute to severe respiratory health damage. Therefore, the understanding of nanoparticle deposition at the tracheobronchial region and alveolar region is important for environmental and occupational health risk assessment. A wide range of studies have reported nanoparticle TD in the nasopharyngeal region of the lung [56,164,165,166]. Most of these studies showed that the DE of smaller diameter particles is higher than for the larger diameter particles in the nasopharyngeal region. However, there is a relatively lower number of studies that have been conducted for nanoparticle TD in the tracheobronchial and terminal airways. The complexity of nanoparticle generation in an experimental study, the difficulty of defining an accurate drug law and the individual correction factor for specific nanoparticles in a CFD study are the main difficulties for nanoparticle TD prediction. A series of studies [167,168,169] have calculated mass transfer and deposition in the upper airways. An experimental study of a human cast reported ultrafine particle deposition and compared it with analytical data [170]. The study concluded that the deposition density at the bifurcation area is 20% greater than in the airway. Zhang and Kleinstreuer [55] studied the airflow structure and nanoparticle deposition for a triple bifurcation model. Euler–Euler (E-E) [55,171,172,173] and Euler–Lagrange (E-L) (Aminfar and Motallebzadeh, 2012; Jayaraju et al. 2008; Kalteh et al. 2011; Longest and Xi, 2007) approaches were commonly used for nanoparticle transport and deposition modelling. In E-L approach, fluid is treated as the primary or continuum phase and discrete or disperse phase is solved by tracing a significant amount of particles or droplets. The key assumption of the E-L approach is the volume fraction of the secondary phase will be lower than the primary phase. On the contrary, the E-E approach treats different phase mathematically as interpenetrating continua.

**Figure 5:**
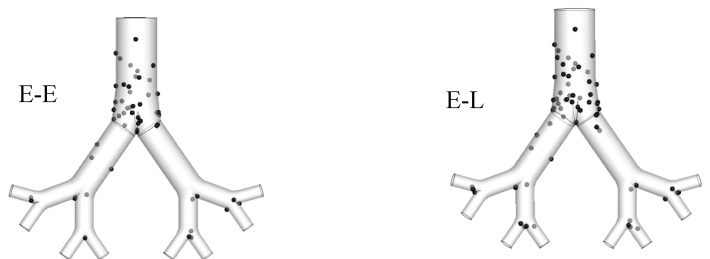
Deposition pattern comparison of 50 nm diameter particle at 25 lpm flow rate. For E-E (**left**) and E-L (**right**) approach.

While the E-E approach neglects the inertia effect on particle transport and considers both the disperse and continuous phase as an interpenetrating field [174], the E-L method usually solves the particle trajectory equations by incorporating all relevant forces [175]. However, the E-E approach is more effective for particle size d_p_ ≤ 100 nm compared to the E-L method, as the inertia of the larger diameter particle influence the overall deposition pattern [99]. The E-E method is more effective than the E-L method for a larger number of particles [176] although the inertia effect has to be considered for larger diameter particles. This study performed the influence of the E-E and E-L methods on nanoparticle deposition for a triple bifurcation airway model (Figure 5). The deposition pattern of a 50-nm diameter particle at 25 lpm flow rate showed no significant difference for both cases.

**Figure 6:**
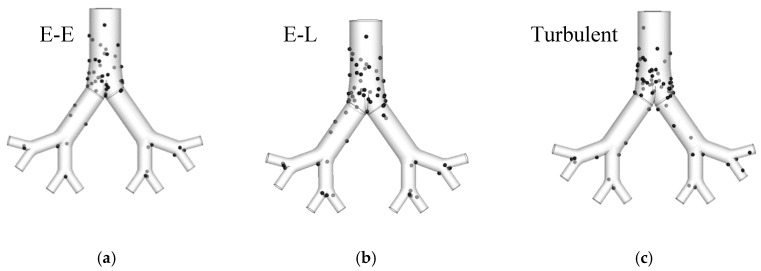
Deposition pattern comparison for different modelling approaches (gray points are the trapped particles on the other side of the airway).

The Euler–Euler approach produced that turbulent effects on nanoparticle deposition were negligible. Turbulent dispersion might occur at the glottis region at a high inspiratory flow rate (Q_in_ ≥ 30 lpm) [55]. However, turbulent fluctuation effects on nanoparticle deposition in the upper airway were found to be not significant [55,58,174]. Similarly, turbulent dispersion effects on nanoparticle deposition in the upper airways were investigated, and the deposition pattern in Figure 6a–c show that turbulent dispersion influence on nanoparticle deposition is also negligible. 

#### 4.2.2. Alveolar Region

The understanding of nanoparticle TD in the acinar region is important for better prediction of respiratory diseases. However, only a few studies have been published that analyzed nanoparticle transport in the acinar region. The published literature reported that alveolar flow is a viscous flow and that it can be kinematically irreversible because of the highly complex structure of the alveoli [177,178,179]. Moreover, the acinar flow can be chaotic which will eventually result in higher deposition [178]. Semmler-Behnke, Kreyling, Schulz, Takenaka, Butler, Henry and Tsuda [177] conducted a numerical investigation of nanoparticle deposition in the acinar region of a rat lung. The study concluded that a smaller nanoparticle size (20 nm) has a higher deposition rate than a larger size nanoparticle (80 nm). Recently, Sturm [180] developed a computer model for nanoparticle deposition in the alveoli. The computer model reported that nanoparticle deposition during a sitting condition varied from 0.1% to 12.4%.

### 4.3. Polydisperse Particle Transport and Deposition

The size distribution of particles from different natural and human-made sources are mostly polydisperse. Despite the polydispersity of atmospheric and therapeutic aerosols, almost all of the in silico, in vivo, and in vitro studies considered monodisperse particle size distribution to illustrate particle transport phenomena in human lung. There is a limited number of in silico and theoretical approaches, which have considered polydisperse particles in their study. There is evidence that the ambient and occupational arrangement of the atmospheric particles are inconsistent in size [181] and these polydispersities have been associated with severe risk for respiratory health [182].

Some theoretical studies (Diu and Yu, 1983; Ferron et al. 1993) have considered polydisperse particles to illustrate regional deposition in human lung and lognormal distribution was used to introduce the polydisperse particles. Rosati, Leith and Kim [181] investigated the deposition pattern of both monodisperse and polydisperse particles in a packed bed. The study reported that the polydisperse particles resulted in higher total deposition compared to a series of monodisperse particles. Recently, Kannan, Przekwas, Singh, Delvadia, Tian and Walenga [88] performed a CFD study to analyze polydisperse particle transport in a ringless trachea model. The seven-generation Zygote5 model used in the study reported the deposition efficiency (DE) for the upper airways. However, the CFD study did not illustrate the size distribution effects on particle transport and deposition.

**Figure 7:**
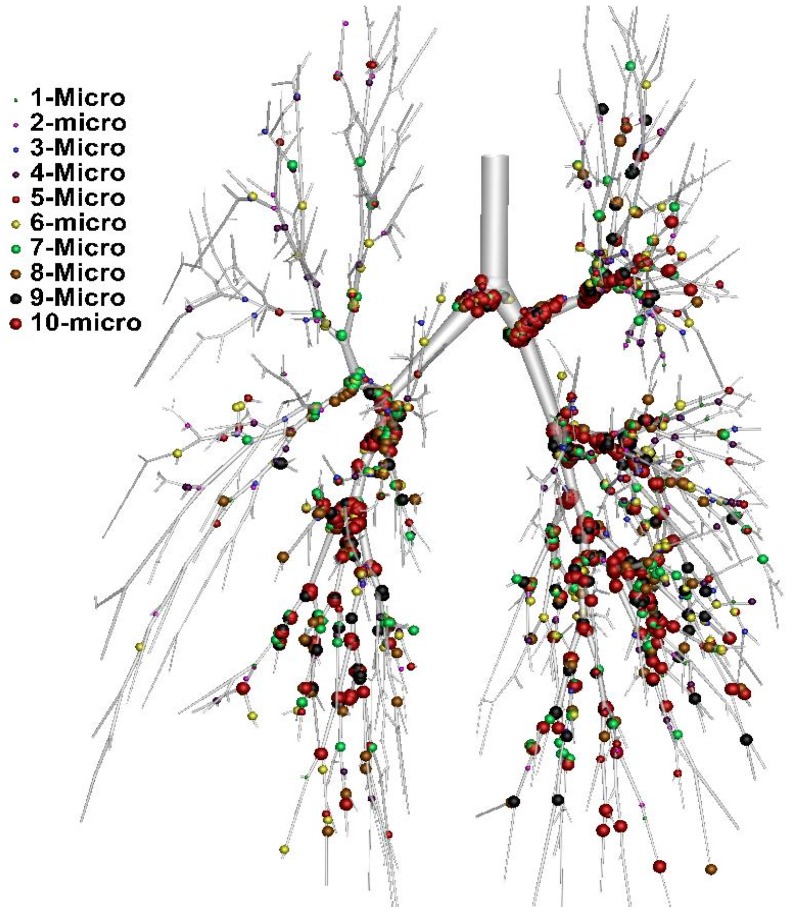
Polydisperse particle deposition pattern in alveoli at 60 lpm flow rate [183].

Recently, Islam, Saha, Gemci, Yang, Sauret and Gu [183] produced a more detailed CFD analysis of polydisperse particle deposition in the terminal airways of a large scale 17-generation model. The 17-generation model considered the entire airway branches. This is the first ever approach to illustrate polydisperse particle deposition in the terminal airways. Figure 7 shows the polydisperse particle deposition at 60 lpm flow rate with the different colours representing the various diameters of particles. The overall deposition pattern shows that larger diameter particles are deposited at the bifurcation areas of the upper airways and smaller diameter particles are deposited at the terminal airways of different lobes.

**Figure 8:**
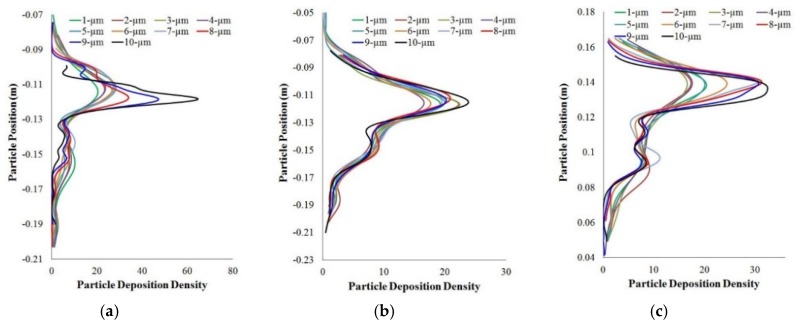

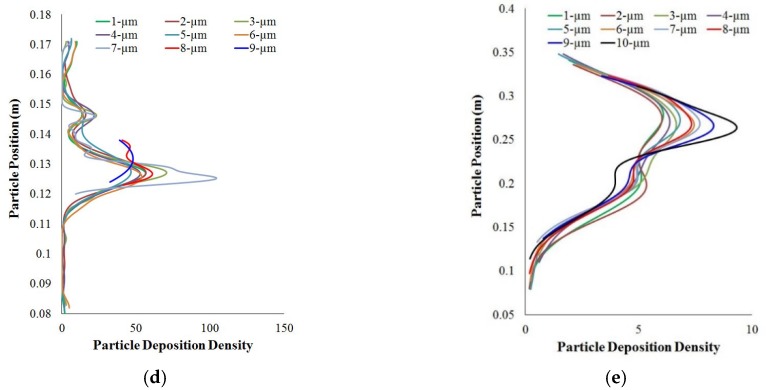
Particle deposition density comparison at different lobes of a 17-generation lung model. (**a**) right upper; (**b**) right middle; (**c**) right lower; (**d**) left upper; (**e**) left lower. Particle deposition density is the number of deposited particles and deposition concentration is calculated along the y-axis of the corresponding lobes [183].

**Figure 9:**
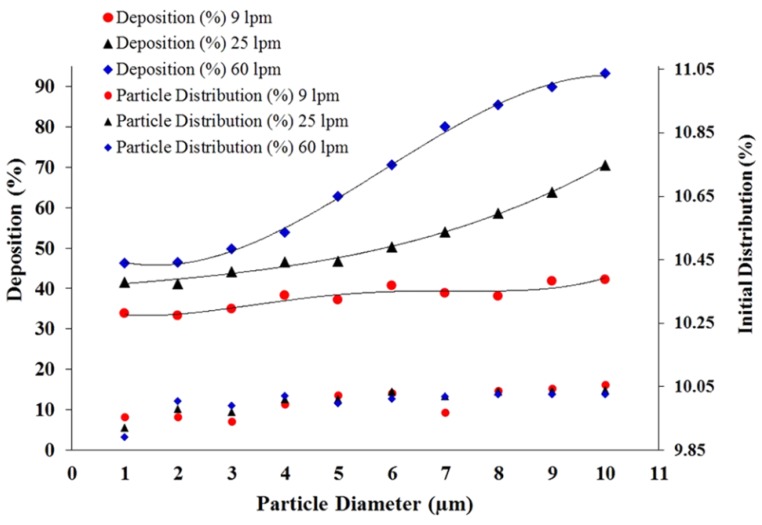
Polydisperse particle initial distribution and deposition efficiency at different flow rates [183].

A more detailed lobe-specific deposition pattern was also investigated in this large scale 17-generation model. Figure 8 shows a deposition density comparison at different lobes (a–e) for size specific polydisperse particles. The lobe specific deposition density curves illustrate the deposition hot spots for different diameter particles at 60 lpm flow rate. This study and highly asymmetric CT-based model help the understanding of realistic particle transport to the terminal airways in human lung. Islam et al. (2018b) also investigated the size-specific particle deposition efficiency for different flow rates. Figure 9 shows the initial distribution and corresponding deposiiton efficiency. The deposition efficiency plot shows an increasing trend with the flow rate and particle diameter.

## 5. Discussions and Perspectives

The review focused on literature findings regarding the modelling of anatomical development, airflow and particle transport or deposition in human lung. All of the presented numerical and experimental studies have improved our understanding of airflow patterns and particle TD in human lung. However, for an even better understanding, some recommendations for future studies are listed below.

### 5.1. Lung Anatomical Model

An anatomical model is the primary component of human lung research. A patient-specific realistic anatomical model would be optimal for respiratory health risk assessments. Almost all of the studies [74,184] to date calculated the airflow characterization and particle transport for an idealized anatomical model. Recently, CT-based realistic models [14] have predicted particle transport in the bifurcating airways. All of these studies considered the oral airways and tracheobronchial airways for their investigation. A 23-generation large-scale idealized human lung model was developed by [185], however the anatomical structure is far away from a realistic model. No CFD study considered a whole lung model. This review recommends that a patient-specific whole lung model should be used for optimum prediction of particle TD.

### 5.2. Numerical Approach

Inhalation and exhalation determine mechanical expansion and contraction of the diaphragm and intercostal muscles of human lung. The two way inhalation and exhalation process influences particle transport to the bifurcating airways [186]. However, almost all of the studies [39,187,188] have considered one-way inhalation for airflow and particle TD prediction. Two-way inhalation and exhalation effect simulation might improve the understanding of particle TD in human lung.

The realistic human lung consists of a complex geometrical structure and airway dimensions are quite smaller for the lower airways than for the upper airways. For a large-scale airway model, the lower airways need to employ a finer computational cell, which overcomes the dimensional scale difference between the upper and lower airways. The finer mesh at the lower airways for a whole lung model significantly increases computational effort. Almost all of the available studies have used single-scale models for airflow and particle deposition prediction. Recently, an image-based multiscale model improved the knowledge of functional relationships and structure of a single bifurcation model [189]. A multi-scale model could be considered to reduce the computational effort for a fine mesh large-scale whole lung model.

### 5.3. Boundary Conditions

Appropriate inlet, outlet, and wall conditions are important boundary factors for human lung modelling. If a whole lung model consists of 23 generations, then there is very little pressure difference at the end of the airway outlet. Almost all of the studies have considered zero and uniform pressure at the outlets of the bifurcating model. Recently, a two-way breathing calculation was performed for a non-uniform pressure outlet, coupled with compliance and resistance [85]. The study reported that non-uniform outlet conditions were capable of reproducing the realistic physiological flow for a diseased lung. Appropriate pressure outlet boundary conditions are important for a more accurate prediction of physiologically realistic flow behaviour, and a health risk evaluation of the abnormal lung.

The wall of the bifurcating airways usually deforms during inhalation and exhalation. Only a number of idealized models consider airway wall deformation [134,190] for airflow characterization and particle TD. There is no published literature which considers the airway wall movement in a digital airway model. Airway wall deformation for CT-based and patient-specific models could be considered for future studies.

### 5.4. Particle–Particle Interaction

Particle–particle interaction effects on particle TD has not been explicitly investigated yet. In reality, during inhalation, particles can collide with each other, although almost all of the published literature did not consider particle–particle collision in their study. However, if a particle suspension entering the tracheobronchial airway is diluted, then direct particle–particle interactions can be neglected. Particle–particle interaction for larger diameter microparticles could influence the deposition pattern in a highly asymmetric realistic model. A detailed analysis of case-specific particle–particle collisions could improve the prediction of realistic deposition patterns in human lung.

## 6. Conclusions

The present study critically reviewed available published literature for the modelling of airflow and particle TD in the extrathoracic and tracheobronchial airways. The review discussed the different laminar and turbulent approaches for transitional flow and particle transport, inlet and outlet conditions, particle–particle interaction, and different particle modelling approaches. Results show that the selection of E-E or E-L modelling approach does not influence smaller diameter particle deposition for smaller flow rates. The results also illustrate that turbulence dispersion does not significantly influence the nanoparticle deposition pattern. When modelling particle–particle interaction a higher DE is achieved than without interaction. However, for modelling particle–particle interaction, particle initial concentrations are important and particle–particle interaction can be neglected if the particle suspension is diluted.

## Figures and Tables

**Table 1:** List of available anatomical models of lung based on CT/MRI images

Author	Model	Anatomy	Generation
Sauret, Halson, Brown, Fleming and Bailey [17]	-	CT	6 and 9
Garrity, Segars, Knisley and Tsui [18]	-	CT	5
McRobbie, Pritchard and Quest [22]	Ex Vivo	MRI	Oropharyngeal Airways
Lin, Tawhai, McLennan and Hoffman [23]	DNS, Subgrid-scale	Multidetector-row CT	Mouth throat to up to 6
Luo and Liu [20]	LRN k-w	CT	5
Walters, Burgreen, Hester, Thompson, Lavallee, Pruett and Wang [19]	Laminar	CT	15
Greenblatt [24]	Theoretical and experimental	CT	9
Rahimi-Gorji, Pourmehran, Gorji-Bandpy and Gorji [25]	k-w SST (steady)	CT	2
Islam, Saha, Sauret, Gu and Ristovski [26]	Laminar, DPM	CT	3
Pourmehran, Rahimi-Gorji, Gorji-Bandpy and Gorji [27]	Turbulent, SST k-w	CT	2
Pourmehran, Gorji and Gorji-Bandpy [28]	k-w Low Reynolds number	CT	6
Miyawaki, Hoffman and Lin [29]	Transitional	CT	5
Miyawaki, Hoffman and Lin [30]	LES	CT	5
Kannan, Chen, Singh, Przekwas, Delvadia, Tian and Walenga [31]	Smagorinsky Model	CT	5
Van de Moortele, Wendt and Coletti [32]	In Vivo, In Vitro	Realistic CT	7
Islam, S. C. Saha and Young [21]	Heliox mixture model	Realistic CT	16

**Table 2:** Flow rate distribution at different lobes of a 17-generation model [99] compared to earlier models (Horsfield et al. (1971) solved analytical equation for flow rate distribution and detail can be found from their study)

Total Flow Rate Distribution (%)			
Region	Cohen et al. (1990) 7.5 (lpm)	Islam et al. (2017c) 7.5 (lpm)	Horsfield et al. (1971)
Left lower	24.5	25.14	24.9
Left upper	14.9	15.34	20.5
Right lower	32.1	36.17	23.2
Right middle	8.3	11.01	9.6
Right upper	20.2	12.48	21.7
Left lung	39.4	40.48	45.4
Right lung	60.6	59.66	54.6

**Table 3:** A list of available literature on particle deposition up to maximum possible generations in human lung

Author	Anatomy	Calculate Particle Deposition?	Generation
[133]	• Non-realistic • Cartilage Ring	√	G-1
[75,134]	• Non-realistic • Weibel’s	√	G0–G3
[15]	• Realistic	√	G0–G6
[135]	• Non-Realistic	√	G0–G6
[136]	• Non-Realistic	√	G0–G7
[137]	• Non-Realistic	√	G0–G9
[74]	• Non-Realistic	√	G0–G10
[95]	• Non-Realistic	√	G0–G15
[138]	• Non-Realistic	√	G0–G16
[92]	• Non-Realistic • Asymmetric	√	G0–17

**Table 4:** Literature on different turbulence modelling for airflow and particle transport

Author	Anatomy	Model	Airflow	Particle Transport and Deposition
Farkas et al. (2006); Zhang, Kleinstreuer, Donohue and Kim [109]; Longest and Vinchurkar, (2007); Zhang et al. (2005b)	• Non-realistic • Weibel’s Model	• Low Reynolds Number (LRN) k-ω turbulence model	√	√
Longest et al. (2016); Xi and Longest [62]; Tian et al. 2015 *	• Realistic Asymmetric	• LRN k-ω turbulence model	√	√
Tian et al. (2017); Farhadi Ghalati et al. (2012); Sohrabi et al. (2017)	• CT-Realistic	• Laminar	√	√
Farkas and Balásházy, (2007); Farkas and Balásházy, (2008); Balásházy et al. (2003); Liu et al. (2002); Longest and Vinchurkar, (2009); Balásházy and Hofmann, (1993); Zhang et al. (2002); Liu et al. (2003a); Nowak, Kakade and Annapragada [72]	• Non-realistic	• Laminar	√	√
Pourmehran, Rahimi-Gorji, Gorji-Bandpy and Gorji [27]; [27,70] **; Sandeau, Katz, Fodil, Louis, Apiou-Sbirlea, Caillibotte and Isabey [37]	• CT-Realistic	• SST k-ω model	√	√
Yousefi et al. [14]; Liu et al. (2007)	• CT-Realistic	• RANS k-ω turbulence method	√	√
Zhang et al. (2004); Matida et al. (2004);	• Idealized	• RANS k-ω turbulence model	√	√
[45,58,106]	• Idealized	• k–ε turbulence model	√	√
Aasgrav et al. (2017); Shih et al. (1995) **	• Realistic	• k-ε turbulence model	√	√
Bernate, Geisler, Padhy, Shaqfeh and Iaccarino [66] **; Cui and Gutheil [64]; Jin, Fan, Zeng and Cen [65]; Gemci, Ponyavin, Chen, Chen and Collins [63] **; Islam, Saha, Sauret, Gemci and Gu [92]	• Realistic • Non-realistic	• LES Turbulence Model	√	√

* No airflow; ** No particle transport and deposition.
